# 659. Epidemiology and Clinical Characteristics of Bacteremia Following Portal Vein Embolization

**DOI:** 10.1093/ofid/ofaf695.214

**Published:** 2026-01-11

**Authors:** Choseok Yoon, Mi Hyeon Park, Eui Jin Chang, SeongMan Bae, Jiwon Jung, Min Jae Kim, Sang-Oh Lee, Sang-Ho Choi, Sung-Han Kim, Yang Soo Kim, Yong Pil Chong

**Affiliations:** Hanyang University Seoul Hosptial, Seoul, Seoul-t'ukpyolsi, Republic of Korea; Asan medical center, Seoul, Seoul-t'ukpyolsi, Republic of Korea; Department of Internal Medicine, Asan Medical Center, Seoul, Korea, Seoul, Seoul-t'ukpyolsi, Republic of Korea; Asan medical center, Seoul, Seoul-t'ukpyolsi, Republic of Korea; Asan Medical Center, Seoul, Seoul-t'ukpyolsi, Republic of Korea; Asan Medical Center, Seoul, Seoul-t'ukpyolsi, Republic of Korea; Asan medical center/Department of Infectious disease, Seoul, Seoul-t'ukpyolsi, Republic of Korea; Asan medical center/Department of Infectious disease, Seoul, Seoul-t'ukpyolsi, Republic of Korea; Asan medical center/Department of Infectious disease, Seoul, Seoul-t'ukpyolsi, Republic of Korea; Asan medical center/Department of Infectious disease, Seoul, Seoul-t'ukpyolsi, Republic of Korea; Asan medical center/Department of Infectious disease, Seoul, Seoul-t'ukpyolsi, Republic of Korea

## Abstract

**Background:**

Portal vein embolization (PVE) is performed to induce liver hypertrophy before major hepatectomy. As an invasive procedure, PVE can lead to complications such as bacteremia, which may result in multi-organ failure. Despite its clinical importance, studies on bacteremia following PVE are limited. This study aimed to examine the epidemiology and clinical characteristics of post-PVE bacteremia.

**Table 1.**
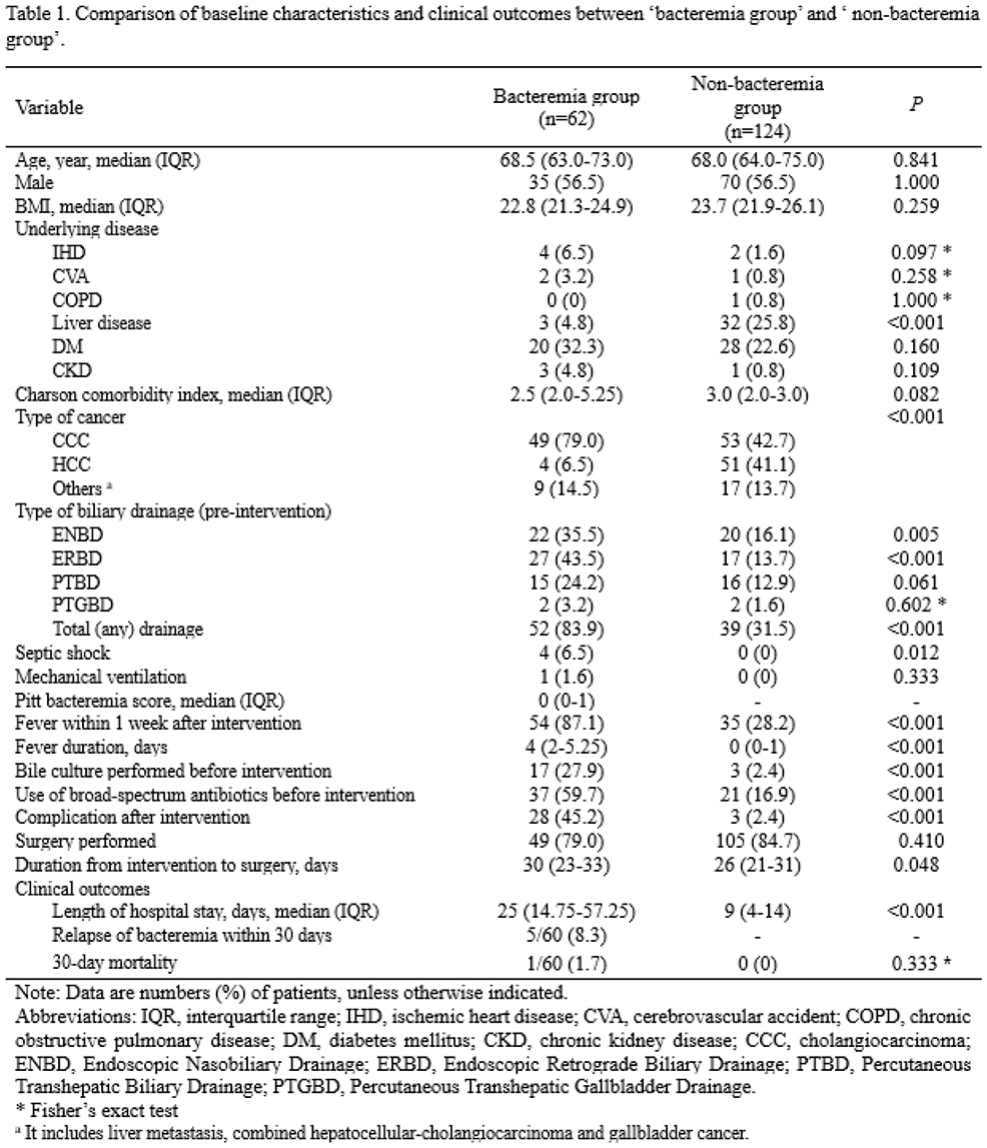
Comparison of baseline characteristics and clinical outcomes between ‘bacteremia group’ and ‘ non-bacteremia group’. Note: Data are numbers (%) of patients, unless otherwise indicated. Abbreviations: IQR, interquartile range; IHD, ischemic heart disease; CVA, cerebrovascular accident; COPD, chronic obstructive pulmonary disease; DM, diabetes mellitus; CKD, chronic kidney disease; CCC, cholangiocarcinoma; ENBD, Endoscopic Nasobiliary Drainage; ERBD, Endoscopic Retrograde Biliary Drainage; PTBD, Percutaneous Transhepatic Biliary Drainage; PTGBD, Percutaneous Transhepatic Gallbladder Drainage. * Fisher’s exact test a It includes liver metastasis, combined hepatocellular-cholangiocarcinoma and gallbladder cancer.

**Table 2.**
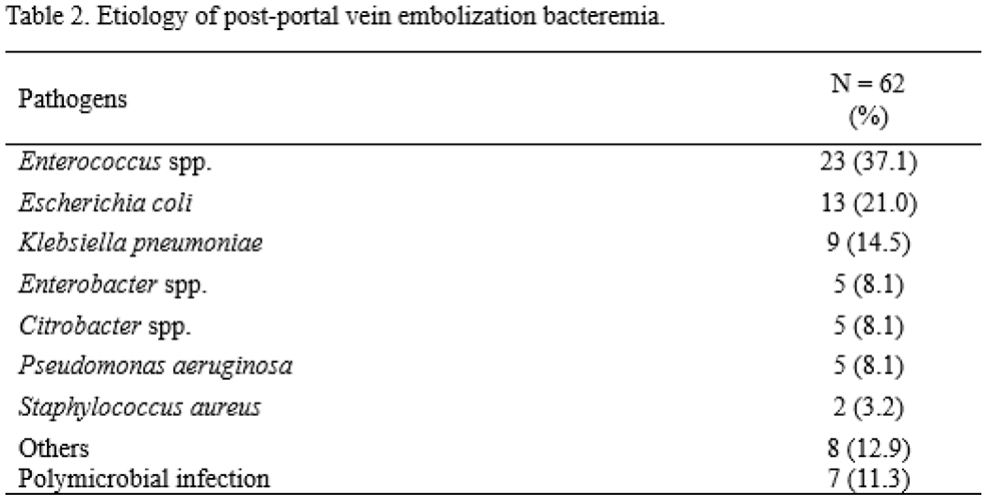
Etiology of post-portal vein embolization bacteremia.

**Methods:**

We retrospectively analyzed adult patients (≥18 years) who developed bacteremia within 30 days of PVE at a single tertiary center (Feb 2014–Mar 2024). Patients with bacteremia after 30 days or post-hepatectomy were excluded. A 1:2 propensity score matching by age, sex, and procedure date was used to select controls without bacteremia. Clinical and microbiological characteristics were compared between bacteremia group and nonbacteremia group.

**Results:**

Among 1,768 PVE patients, 62 (3.5%) developed bacteremia. Of these, 48 (77.4%) had no other major complications, while 14 (22.6%) had. *Enterococcus* spp. (35.5%) was the most common pathogen followed by *Escherichia coli* (21.0%). Extended-spectrum cephalosporins were most frequently used before procedure (77.8%), while beta-lactam/beta-lactamase inhibitors were most frequently used after bacteremia (51.7%) as emprical antibiotics. Median time from PVE to bacteremia was 3 days (IQR 1–7); median bacteremia duration was 1 day (IQR 1–1). Compared to controls, the bacteremia group had more cholangiocarcinoma and fewer hepatocellular carcinoma cases (P < 0.001), and more frequent pre-procedural biliary drainage (83.9% vs. 31.5%, P < 0.001), especially ENBD and ERBD. Rates of post-PVE fever (87.1% vs. 28.2%), pre-procedure broad-spectrum antibiotic use (59.7% vs. 16.9%), and any complications (45.2% vs. 2.4%) were significantly higher (all P < 0.001) in bacteremia group than those of nonbacteremia group. Additionally, time to surgery (30 vs. 26 days, P = 0.048) and hospital stay (25 vs. 9 days, P < 0.001) were longer in the bacteremia group than those of nonbacteremia group.

**Conclusion:**

Post-PVE bacteremia was more frequent in patients with cholangiocarcinoma, particularly those undergoing ENBD or ERBD. *Enterococcus* spp. was the most frequent causative organism.

**Disclosures:**

All Authors: No reported disclosures

